# Influence of sex and age on inferior vena cava diameter and implications for the implantation of vena cava filters

**DOI:** 10.1590/1677-5449.202101471

**Published:** 2022-11-04

**Authors:** Ana Carolina Corrêa Franco, Lillian dos Santos Carneiro, Reinaldo Sérgio Monteiro Franco, Adenauer Marinho de Oliveira Góes

**Affiliations:** 1 Centro Universitário do Estado do Pará - CESUPA, Belém, PA, Brasil.

**Keywords:** embolism and thrombosis, vena cava filter, tomography, inferior vena cava, anatomy

## Abstract

**Background:**

Measuring the venous diameter and choosing a compatible vena cava filter are essential to reduce the risk of complications resulting from implantation of these devices. However, there is little information on how the diameter of the inferior vena cava varies with sex and age.

**Objectives:**

To determine the influence of patients’ gender and age on their inferior vena cava diameter and the suitability of the different models of available filters.

**Methods:**

Retrospective analytical study based on computed tomography images. The diameter of the inferior vena cava was measured at 3 points: above the confluence of the common iliac veins, below the renal veins, and midway between these two points (cranial point, caudal point, and midpoint) using Arya® and Carestream PACS® software. The results were classified by sex and age groups.

**Results:**

CT scans of 417 patients were analyzed: 245 women and 172 men. The diameters at the midpoint and caudal point were, respectively, 19.1 mm and 20.6 mm in women from 81 to 92 years old and were statistically smaller (p< 0.05) when compared to women aged 19 to 40 years (midpoint: 22.7 mm; caudal point: 23 mm). Similar results were seen in men. Venous diameters at the cranial and caudal points in patients aged from 51 to 70 years were statistically larger in men (cranial point: 24.4 mm; caudal point:22.3 mm) than in women (cranial point: 22.6 mm; caudal point:20.8 mm) (p< 0.05).

**Conclusions:**

A smaller diameter was found for the inferior vena cava in older patients of both sexes and the rate of diameter change was similar among men and women.

## INTRODUCTION

Vena cava filter (VCF) placement is recommended in patients with venous thromboembolism (VTE) who have contraindications to anticoagulation, such as those with active bleeding or thrombocytopenia.^1^^-^6

There are more than ten different models of VCF available on the market, indicated for veins with diameters ranging from 14 to 35 mm. However, complications resulting from implanting these devices include inferior vena cava (IVC) perforation and filter migration.^6^^-^14 To reduce the risk of these complications, it is essential to measure the IVC diameter using tomography, Doppler ultrasonography, or phlebography, in order to select a compatible filter.

Although removable filters do exist, these devices are frequently not removed. However, there is little information on how IVC diameters vary as the patients get older. A review of literature on the subject identified just one article,^15^ which used echocardiograms and demonstrated that intrapericardial IVC diameter tends to reduce as patients age. However, no similar studies regarding the infrarenal segment were found.

The aim of this study was to determine the influence of sex and age of patients on the diameter of the infrarenal segment of the IVC and the suitability of different VCF models according to the variation in this anatomic parameter.

## Methods

This was a retrospective analytical study, approved by the Research Ethics Committee (protocol number 4.448.908), analyzing computed tomographies (CTs) in order to measure IVC diameters.

The tomographies analyzed were performed from January 2015 to January 2021 on GE VCT, 64 channel scanners (GE HealthCare, Chicago, IL, USA) or on Siemens Somatom Scope (Siemens Healthcare, Erlangen, Germany), 16 channel scanners, using Picture Archiving Communication System (PACS) Aurora Arya (PIXEON, São Caetano do Sul, SP, Brazil) version 20.10.1 and Carestream Vue PACS (Carestream Health, Rochester, NY, USA) version 12.1.5.0417 software.^16^^,^^17^

Sample size was calculated using the Fontelles 2012 rule, by which a minimum sample of 348 examinations was considered representative.^18^

**Figure 1:**
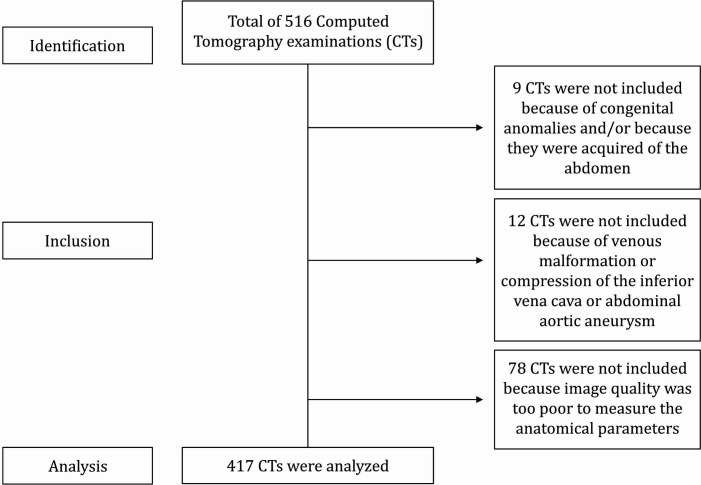
Flow diagram of examinations analyzed in the study.

Patients of both sexes, aged 19 years or older, were included. CTs were excluded if they showed congenital anomalies, showed venous malformations and extrinsic compression on the IVC, and/or when the definition of the images did not enable measurement of the anatomic parameters investigated (Figure 1).

**Figure 2:**
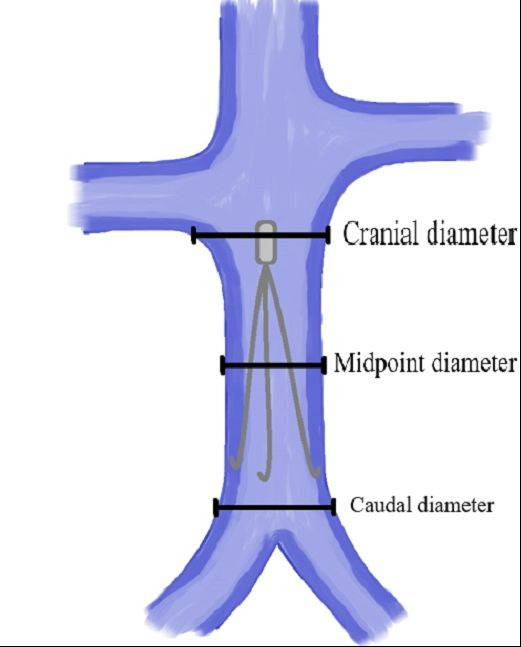
Diagram illustrating the points of measurement of the diameters of the inferior vena cava.

**Figure 3:**
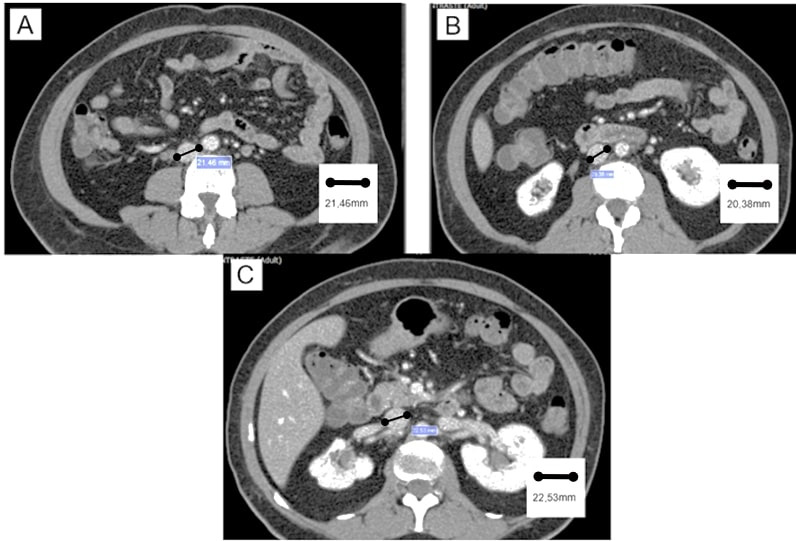
Measurement of the points on computed tomography images. (A) Measurement of caudal point; (B) Measurement of midpoint; and (C) Measurement of cranial point.

The IVC diameter was measured on axial slices at three points: caudal diameter (immediately above the confluence of the common iliac veins), cranial diameter (immediately below the most caudal renal vein), and diameter at the midpoint (halfway between the cranial and caudal measurement points) (Figures 2 and 3).

The variables were analyzed according to sex and by distribution in the following age ranges: 19 to 40, 41 to 50, 51 to 60, 61 to 70, 71 to 80, and 81 to 92 years.

Quantitative variables were expressed as minimum, maximum, mean, and standard deviation, and qualitative variables were expressed as frequency and percentage. Numerical variables were compared between two groups using Student’s *t* test or the Mann-Whitney test, its nonparametric equivalent. Numerical variables were compared between more than two groups using analysis of variance (ANOVA) or the Kruskal-Wallis test, its nonparametric equivalent.

The rate of diameter narrowing, in male and female patients, was calculated by subtracting the mean diameter for the oldest age range (81 to 92 years) from the mean diameter for the youngest age range (19 to 40 years) and multiplying by 100. These rates were compared by sex using Student’s *t* test. The normality of data distribution was tested using the Shapiro-Wilk test. Results with p ≤ 0.05 (bilateral) were considered statistically significant.

Microsoft Excel^®^ 2010 (Microsoft Corporation, Redmond, WA, USA) and BioEstat 5.5 (Sartorius, Gottingen, Germany) were used to tabulate data, perform statistical tests, and plot graphs.^19^^,^^20^

## Results

After application of the inclusion and exclusion criteria, the final sample comprised 417 patients, 245 (58.8%) of whom were female. Mean age was 57.8±13.6 years, ranging from 19 to 92 years. There was a statistically significant (†) predominance of female patients in the age group from 41 to 50 years and of male patients in the age group from 61 to 70 years (Table 1). Table 2 lists mean age by sex, with no significant difference.

**Table 1:** Distribution of patients by sex and age group.

**Variable**	**Total (n=417) AF (Fr%)**	**Females (n=245) AF (Fr%)**	**Males (n=172) AF (Fr%)**	***p*-value**
**Age**				0.031^*^
19 to 40 years	55 (13.2%)	38 (15.5%)	17 (9.9%)	
41 to 50 years	64 (15.3%)	45 (18.4%)†	19 (11.0%)	
51 to 60 years	108 (25.9%)	65 (26.5%)	43 (25.0%)	
61 to 70 years	112 (26.9%)	53 (21.6%)	59 (34.3%)†	
71 to 80 years	64 (15.3%)	36 (14.7%)	28 (16.3%)	
81 to 92 years	14 (3.4%)	8 (3.3%)	6 (3.5%)	

Variables are expressed as n (%). AF: absolute frequency of patients in each age group (overall and by sex); Fr%: relative frequency of patients in each age group.

* Chi-square test of independence/analysis of residuals;

† p ≤ 0.05.

**Table 2:** Mean age by sex.

**Age**	**Total (n=417)**	**Females (n=245)**	**Males (n=172)**	***p*-value**
	57.7±13.6	56.1±14.1	60.1±12.5	0.003^*^

Ages are expressed as: mean ± standard deviation.

* Mann-Whitney test.

The IVC diameter at the caudal measurement point reduced significantly with advancing age in both sexes (men: p = 0.02; women: p < 0.001). At this measurement point, the IVC had a statistically larger caliber in men than in women in two of the age groups: 51 to 60 years (p = 0.003) and 61 to 70 years (p < 0.001).

Men and women exhibited progressively smaller diameters at the midpoint of the infrarenal vena cava (both sexes: p < 0.001), but at this measurement point there were no significant differences in diameter between the sexes in any of the age groups.

At the most cranial measurement point, the IVC caliber was statistically larger in men than women in the following age groups: 41 to 50 years (mean diameter of 24.6 mm in men and 22.7 mm in women; p = 0.01); 51 to 60 years (mean diameter of 24.8 mm in men and 22.8 mm in women; p = 0.03); and 61 to 70 years (mean diameter of 24 mm in men and 22.4 mm in women; p = 0.03).

**Figure 4:**
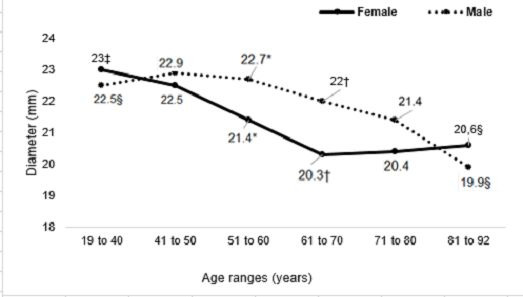
Mean diameters of the vena cava at the caudal measurement point in both sexes, by age groups. *p = 0.003; Student’s *t*. †p < 0.001; Student’s *t*. ‡p < 0.001; Kruskal-Wallis. §p = 0.02; analysis of variance.

**Figure 5:**
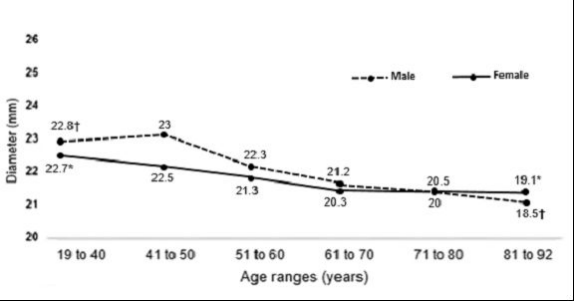
Mean diameters of the vena cava at the midpoint measurement in both sexes, by age groups. *and †p < 0.001; analysis of variance.

**Figure 6:**
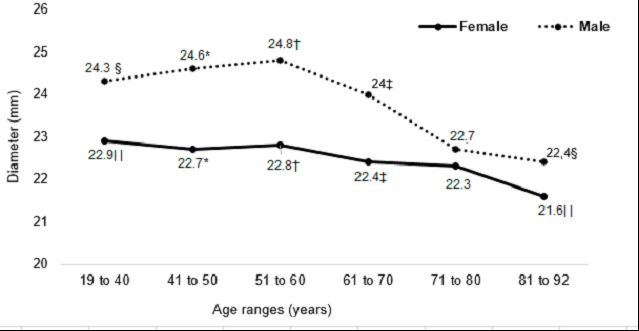
Mean diameters of the vena cava at the cranial measurement point in both sexes, by age groups. *p = 0.01; Student’s *t* test. † and ‡p = 0.03; Student’s *t* test. § p = 0.83; Kruskal-Wallis. || p = 0.06; Kruskal-Wallis.

The mean IVC diameters of patients of both sexes in the different age groups measured at the caudal point, midpoint, and cranial point are illustrated, respectively, in Figures 4, 5, and 6.

**Figure 7:**
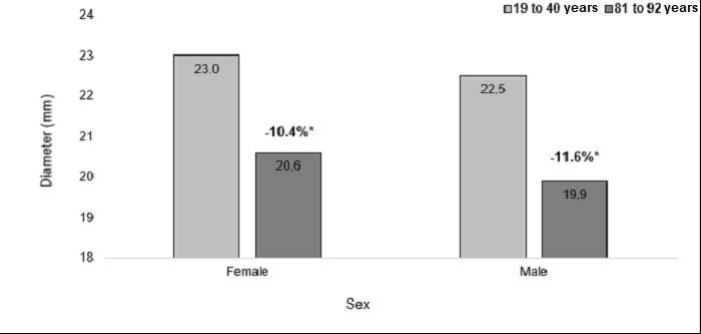
Rates of change in diameter at the caudal measurement point, in both sexes. *p = 0.54; Student’s *t* test.

**Figure 8:**
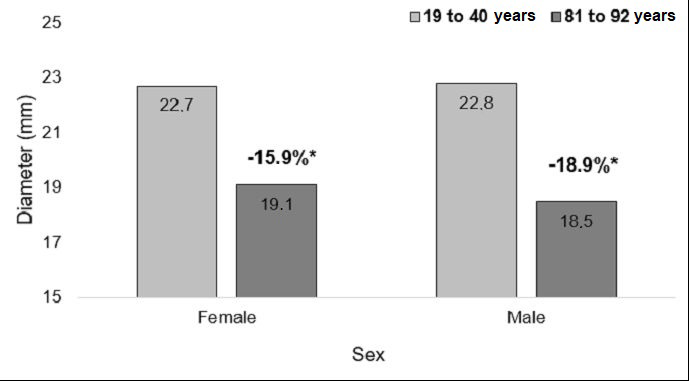
Rates of change in diameter at the midpoint measurement, in both sexes. *p = 0.10; Student’s *t* test.

**Figure 9:**
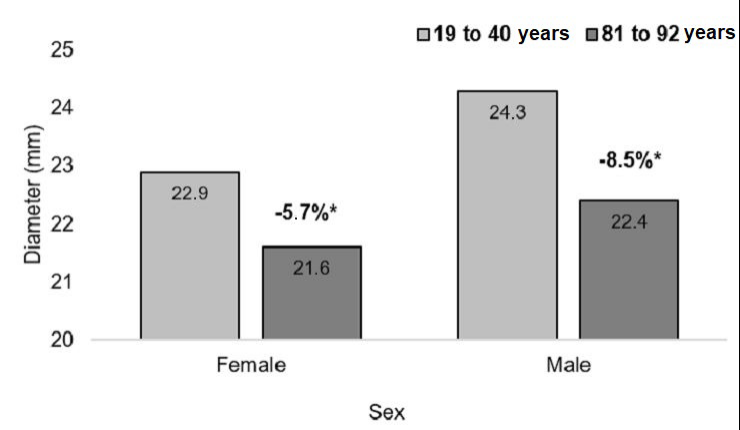
Rates of change in diameter at the cranial measurement point, in both sexes. *p = 0.48; Student’s *t* test.

Among the women, the mean diameters measured at the caudal point, midpoint, and cranial point were, respectively, 22.9 mm, 22.7 mm, and 23 mm in the youngest age group (19 to 40 years) and 21.6 mm, 19.1 mm, and 20.6 mm in the oldest age group (81 to 92 years). Among the men, the mean diameters in the youngest age group were, respectively, 22.4 mm, 22.8 mm, and 22.5 mm and mean diameters in the oldest age group were 22.4 mm, 18.5 mm, and 19.9 mm. The rates of change of the mean IVC diameters at the three measurement points are illustrated in Figures 7, 8, and 9.

Although smaller diameters were identified in both women and older men, the rate of change in diameters at the cranial, mid and caudal points was similar between the sexes (p = 0.54, p = 0.10, and p = 0.48).

## Discussion

The first VCF, the Mobin Uddin model, was developed in 1969. Since then, these filters have undergone many modifications to increase their efficiency and reduce the incidence of complications.^21^^-^23

**Figure 10:**
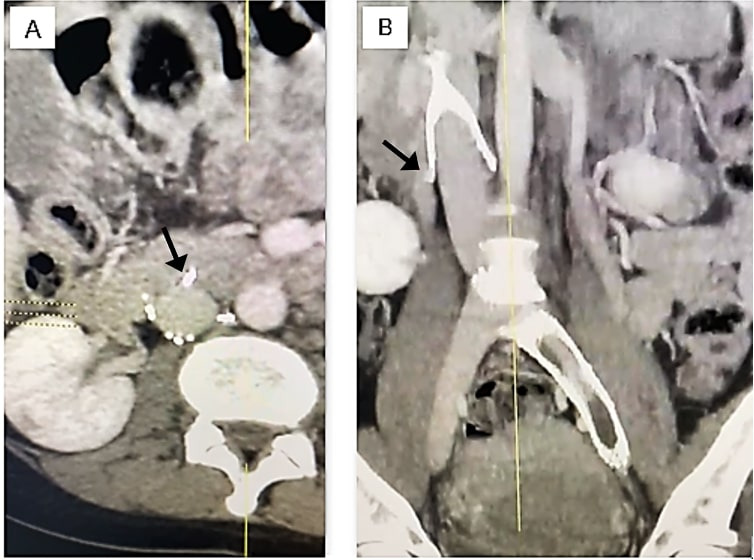
Vena cava filter perforating the inferior vena cava. (A) Axial computed tomography; (B) Coronal computed tomography. Arrowhead: filter securing struts perforating the wall of the vena cava. Source: Authors’ personal archives.

The chosen VCF must fit the diameter of the patient’s IVC in order to avoid complications such as IVC, and adjacent structures, perforation (Figure 10), filter migration or thrombosis, or device embolization.^8^^-^14 Currently, at least 14 models of VCF are available in Brazil. Their characteristics, including compatibility with IVC diameter, are listed in Table 3.

**Table 3:** Types of vena cava filter and their characteristics.

**Product**	**Country**	**Manufacturer**	**Material**	**Shape**	**Diameter**	**Removal**
**GREENFIELD®**	United States	Boston	Steel/titanium	Conical	≤ 28mm	Permanent
**GUNTHER®**	United States	Cook	Conichrome	Conical	≤ 30mm	≤ 3 weeks^*^
**BIRD’S NEST®**†	United States	Cook	Conichrome	Nest	35-40mm	Permanent
**CELECT®**	United States	Cook	Conichrome	Conical	≤ 30mm	≤ 3 months*
**VENATECH CONVERTIBLE®**	France/United States	BBraun	Phynox	Conical	≤ 32mm	≤ 4 weeks*
**VENATECH RETRIEVABLE®**	France/United States	BBraun	Phynox	Conical	14-28mm	≤ 12 weeks*
**VENATECH LP®**	France/United States	BBraun	Phynox	Conical	28-35mm	Permanent
**VENATECH LGM®**	France/United States	BBraun	Phynox	Conical	≤ 28mm	Permanent
**TEMPOFILTER II®**	France/United States	BBraun	Phynox	Conical	≤ 32mm	Permanent
**SIMON®**	United Kingdom	Bard	Nitinol	Mushroom	≤ 32mm	≤ 12 weeks*
**TRAPEASE®**	United States	Cordis	Nitinol	Trapezoid	≤ 30mm	Permanent
**OPTEASE®**	United States	Cordis	Nitinol	Trapezoid	≤ 30mm	≤ 21dias*
**ALN®**	France	ALN	Steel	Conical	≤ 32mm	≤ 25 months*
**ELLA®**	Czech Republic	ELLA	Steel	Conical	18-35mm	≤ 12 days*

* Time defined by the manufacturer as the maximum before retrieval of the filter;

† Not registered for use for Brazil. Source: National Agency for Sanitary Vigilance (Agência Nacional de Vigilância Sanitária - ANVISA)^24^.

The classic topography for VCF placement is the infrarenal segment, where it is recommended that the top of the filter should be located immediately below the most caudal renal vein (matching the cranial diameter measurement point in this study), so that renal drainage is not compromised if retained thrombi obstruct the filter.

A review of the literature did not find any studies that demonstrated the trend for older patients to have smaller IVC diameters in the infrarenal segment, as was demonstrated in the present study, or that studies discussing the possible correlation of IVC narrowing and development of late FVC complications.

In 2010, Masugata et al.^15^ published results of analyses of the diameter of the intrapericardial segment of the IVC and demonstrated that the trend in older patients is for the cava wall to contract, leading to a progressive narrowing of the lumen in this topography. They suggested that these variations occurred because of reduced right atrial pressure and IVC compliance as age increases.

In our study, patients were distributed by decades of age, as in the study conducted by Masugata et al.^15^ However, since imaging exams are more often ordered for older patients, we combined patients aged 19 to 40 years into one group, so that the age groups had comparable numbers of patients. In both studies, the one by Masugata et al.^15^ and the present one, the relationship between IVC diameters and patient age was analyzed at a single time of observation. The ideal methodology, although unfeasible because of countless limitations, would involve following the changes in venous diameter over the course of decades in a significant number of patients.

Despite these limitations, the conclusions of both studies converge on a tendency for IVC diameter to reduce as patients get older. This phenomenon is the opposite of what occurs with the abdominal aorta, the diameter of which trends to increase, as demonstrated in a previously published study conducted by our research group.^25^

As people age, the reduction in collagens and the effects of free radicals are undoubtedly systemic and manifest in both veins and arteries, but with opposite consequences in these major abdominal vessels.^26^^-^29 This difference is probably because whereas the physiology of the arterial system is more based on “pressure”, the function of the venous system is based on “compliance”.^30^ As patients age, there is an increased predisposition to peripheral blood stasis, even influencing the development of venous insufficiency.^31^ As such, in theory, the IVC would store a progressively lower volume of blood over time. As wall distension reduces and wall elasticity is lost, its diameter reduces progressively. However, studies to confirm these theories have not yet been performed.

Although all filters are capable of adapting to different vein diameters, this adaptive capacity is limited and related to the filter’s ability to fit the IVC diameter at deployment. It is uncertain how the structure of the device would accommodate possible reductions in the caliber of a vein with progressively less elastic walls and this may be associated with late perforation of the IVC and with a consequent risk of injury to adjacent structures.^9^^-^11^,^^14^

According to our results, the majority of filters are compatible with IVC infrarenal diameters of adult men and women. However, the progressive reduction of IVC diameters could interfere with the compatibility of the Bird’s nest**®** (Cook Medical, Bloomington, IN, USA) and VenaTech® LP (B. Braun Sharing Expertise, Melsungen, Germany) models, because they are indicated for diameters above 28 mm.

Very often, the filter implanted is the one that is available, i.e. the filter that has been approved by a health insurance plan or the one available at a given public hospital, which may increase the risk of complications. For example, if a given filter compatible with vena cava diameters ranging from 28 to 35 mm (Table 3) is implanted in a woman younger than 40 years old, since the mean diameter at the midpoint of the infrarenal segment for this patient profile was found to be 22.8 mm, the risk of acute complications would already be potentially high; but our theory is that the risk would become even higher as the patient grows older, since, over the next four decades, her vena cava diameter could reduce by 15.9%, increasing the risk of IVC perforation.

The practical implication of the present study is that, whenever possible, the chosen VCF should be a removable model and it also highlights the importance of individual assessment of the vena cava diameter before VCF deployment.^32^

The limitations of this study include its retrospective nature; for example, it was not possible to collect data on the height and weight of patients which, hypothetically, could be related to variations in IVC diameter. Additionally, although a sample size calculation was performed and we did obtain a total patient sample that exceeded the minimum sample size, the distribution of the numbers of patients in each age group was by convenience, according to the availability of tomographic examinations performed. Additional research regarding the topic is suggested.

## CONCLUSIONS

The diameter of the infrarenal segment of the IVC was smaller in older patients, both among men and among women, and the sex of patients did not have a significant influence on the rate of diameter narrowing.

The majority of VCF models are compatible with the infrarenal diameters of the IVCs of adult men and women aged 20 to 92 years.
