# Supplementary material for: Influence of sex and age on inferior vena cava diameter and implications for the implantation of vena cava filters
Source: J Vasc Bras. 2022 Nov 4;21:e20210147. doi: 10.1590/1677-5449.202101471 (PMC9639577; doi:10.1590/1677-5449.202101471)
Supplement: Translation

# Translation: Influência do sexo e da idade sobre o diâmetro da cava inferior e implicações para o implante de filtros de veia cava

**DOI:** 10.1590/1677-5449.202101472

**Authors:** Ana Carolina Corrêa Franco, Lillian dos Santos Carneiro, Reinaldo Sérgio Monteiro Franco, Adenauer Marinho de Oliveira Góes

**Affiliations:** 1 Centro Universitário do Estado do Pará – CESUPA, Belém, PA, Brasil.

**Keywords:** embolia e trombose, filtro de veia cava, tomografia, veia cava inferior, anatomia

## Abstract

**Contexto:**

A aferição do diâmetro venoso e a escolha de um filtro de veia cava compatível são fundamentais para diminuir o risco de complicações decorrentes do implante desses dispositivos. Entretanto, são escassas as informações sobre como o diâmetro da cava inferior varia de acordo com o sexo e a idade.

**Objetivos:**

Determinar a influência do sexo e da idade dos pacientes sobre o diâmetro da cava inferior e a adequação dos diferentes modelos de filtro disponíveis.

**Métodos:**

Estudo analítico retrospectivo, realizado a partir de imagens de tomografia computadorizada. O diâmetro no segmento infrarrenal da veia cava inferior foi aferido em três pontos (cranial, médio e caudal). Os resultados foram classificados de acordo com o sexo e as faixas etárias.

**Resultados:**

Foram analisadas tomografias de 417 pacientes: 245 mulheres e 172 homens. Os diâmetros nos pontos médio e caudal foram, respectivamente, 19,1 mm e 20,6 mm em mulheres de 81 a 92 anos, sendo estatisticamente menores (p < 0,05) quando comparados aos de mulheres com idade entre 19 e 40 anos (diâmetro no ponto médio: 22,7 mm; diâmetro no ponto caudal: 23 mm). Resultados semelhantes foram observados em homens. Os diâmetros venosos nos pontos cranial e caudal foram estatisticamente maiores em homens (ponto cranial: 24,4 mm; ponto caudal: 22,3 mm) do que em mulheres (ponto cranial: 22,6 mm; ponto caudal: 20,8 mm) em pacientes com idade entre 51 e 70 anos (p < 0,05).

**Conclusões:**

O diâmetro da veia cava inferior foi menor em pacientes com idade mais avançada em ambos os sexos, e a taxa de variação do diâmetro foi semelhante entre homens e mulheres.

## INTRODUÇÃO

O implante de filtros de veia cava (FVC) é recomendado em pacientes com tromboembolismo venoso (TEV) que apresentem contraindicação à anticoagulação, como aqueles com sangramento ativo ou plaquetopenia^1^^-^6.

Há mais de dez modelos de FVC disponíveis no mercado, indicados para veias com diâmetro entre 14 e 35 mm. Entretanto, entre as complicações advindas do implante desses dispositivos, encontram-se a perfuração da veia cava inferior (VCI) e a migração do filtro^6^^-^14. A fim de diminuir o risco dessas complicações, é fundamental que o diâmetro da VCI seja aferido por meio de tomografia, ultrassonografia com Doppler ou flebografia, para que seja implantado um filtro compatível.

Apesar de haver filtros removíveis, muitos pacientes permanecem com este dispositivo ao longo da vida. Todavia, são escassas informações sobre como o diâmetro da VCI varia conforme os pacientes envelhecem. Durante a revisão da literatura sobre o tema, foi identificado um único artigo^15^ no qual, utilizando ecocardiograma, demonstrou-se que o diâmetro do segmento intrapericárdico da VCI tende a diminuir conforme os pacientes envelhecem. No entanto, estudos semelhantes para o segmento infrarrenal não foram localizados.

O objetivo deste estudo foi determinar a influência do sexo e da idade dos pacientes sobre o diâmetro do segmento infrarrenal da VCI e a adequação dos diferentes modelos de FVC de acordo com a variação desse parâmetro anatômico.

## MÉTODOS

Tratou-se de um estudo analítico retrospectivo, aprovado pelo Comitê de Ética em Pesquisa (parecer n.º 4.448.908), que avaliou tomografias computadorizadas (TC) a fim de medir o diâmetro da VCI.

Foram avaliados exames realizados entre janeiro de 2015 e janeiro de 2021 em tomógrafos VCT GE, 64 canais (GE HealthCare, Chicago, IL, EUA), e no tomógrafo Siemens Somatom Scope (Siemens Healthcare, Erlangen, Alemanha), 16 canais, utilizando os programas Picture Archiving Communication System (PACS) Aurora Arya (PIXEON, São Caetano do Sul, SP) versão 20.10.1 e Carestream Vue PACS (Carestream Health, Rochester, NY, EUA) versão 12.1.5.0417^16^^,^^17^.

Realizou-se cálculo de tamanho amostral pela regra de Fontelles 2012, com uma amostra mínima de 348 exames tendo sido considerada representativa^18^.

Foram incluídos exames de pacientes de ambos os sexos, com idade igual ou superior a 19 anos. Excluíram-se TCs evidenciando anomalias congênitas ou adquiridas do abdome, malformações venosas e compressão extrínseca sobre a VCI e/ou quando a definição das imagens não permitiu a mensuração dos parâmetros anatômicos pesquisados (Figura 1).

**Figura 1:** Diagrama de fluxo dos exames analisados no estudo.

O diâmetro da VCI em cortes axiais foi aferido em três pontos: diâmetro caudal (imediatamente acima da confluência das veias ilíacas comuns), diâmetro cranial (imediatamente abaixo da desembocadura da veia renal mais caudal) e diâmetro no ponto médio (à meia distância entre os pontos de medida cranial e caudal) (Figuras 2 e 3).

**Figura 2:** Esquematização dos pontos de aferição dos diâmetros da veia cava inferior.

**Figura 3:** Medição dos pontos através da tomografia computadorizada. (A) Medida do ponto caudal; (B) medida do ponto médio; e (C) medida do ponto cranial.

As variáveis foram analisadas de acordo com o sexo e a distribuição nas seguintes faixas etárias: 19 a 40, 41 a 50, 51 a 60, 61 a 70, 71 a 80 e 81 a 92 anos.

As variáveis quantitativas foram descritas por mínimo, máximo, média e desvio-padrão, e as variáveis qualitativas foram descritas por frequência e percentagem. Para comparar uma variável numérica entre dois grupos, foi utilizado o teste *t* de Student ou o teste de Mann-Whitney, seu equivalente não paramétrico. Para comparar uma variável numérica entre mais de dois grupos, utilizou-se a análise de variância (ANOVA) ou o teste de Kruskal-Wallis, seu equivalente não paramétrico.

As taxas de variação dos diâmetros de acordo com o sexo foram calculadas subtraindo-se o diâmetro médio na faixa etária superior (81 a 92 anos) do diâmetro médio na faixa etária inferior (19 a 40 anos) e multiplicando-se por 100. Essas taxas foram comparadas entre os sexos por meio do teste *t* de Student. A normalidade dos dados foi testada pelo teste de Shapiro-Wilk. Os resultados com p ≤ 0,05 (bilateral) foram considerados estatisticamente significativos.

Para tabulação dos dados, realização dos testes estatísticos e confecção de gráficos e tabelas, foram utilizados os programas Microsoft Excel® 2010 (Microsoft Corporation, Redmond, WA, EUA) e BioEstat 5.5^19^^,^^20^ (Sartorius, Gottingen, Alemanha).

## RESULTADOS

Após aplicados os critérios de inclusão e exclusão, a amostra resultou em 417 pacientes, entre os quais 245 (58,8%) eram do sexo feminino. A média de idade foi de 57,8±13,6 anos, variando de 19 a 92 anos. Observou-se predominância estatisticamente significativa (†) de pacientes do sexo feminino na faixa etária de 41 a 50 anos e de pacientes do sexo masculino na faixa etária de 61 a 70 anos (Tabela 1). A Tabela 2 apresenta a idade média segundo o sexo, não tendo ocorrido diferença significativa.

**Tabela 1:** Distribuição dos pacientes segundo sexo e faixa etária.

**Variável**	**Total (n=417) Fa (Fr%)**	**Feminino (n=245) Fa (Fr%)**	**Masculino (n=172) Fa (Fr%)**	**p-valor**
**Idade**				0,031*
19 a 40 anos	55 (13,2%)	38 (15,5%)	17 (9,9%)	
41 a 50 anos	64 (15,3%)	45 (18,4%)†	19 (11,0%)	
51 a 60 anos	108 (25,9%)	65 (26,5%)	43 (25,0%)	
61 a 70 anos	112 (26,9%)	53 (21,6%)	59 (34,3%)†	
71 a 80 anos	64 (15,3%)	36 (14,7%)	28 (16,3%)	
81 a 92 anos	14 (3,4%)	8 (3,3%)	6 (3,5%)	

As variáveis são representadas como n (%). Fa: frequência absoluta de pacientes em cada faixa etária (de maneira global e separados por gênero); Fr%: frequência relativa dos pacientes em cada faixa etária.

* Teste do qui-quadrado de independência/análise de resíduos;

† p ≤ 0,05.

**Tabela 2:** Idade média segundo o sexo.

**Idade**	**Total (n=417)**	**Feminino (n=245)**	**Masculino (n=172)**	**p-valor**
Geral	57,7±13,6	56,1±14,1	60,1±12,5	0,003*

As idades são representadas como: média ± desvio-padrão.

* Teste de Mann-Whitney.

O diâmetro da VCI no ponto de aferição caudal reduziu significativamente com o avançar da idade em ambos os sexos (homens: p = 0,02; mulheres: p < 0,001). Nesse segmento, a VCI foi estatisticamente mais calibrosa em homens do que em mulheres em duas faixas etárias: 51 a 60 anos (p = 0,003) e 61 a 70 anos (p < 0,001).

Homens e mulheres apresentaram diâmetro progressivamente menor no segmento médio da cava infrarrenal (ambos os sexos: p < 0,001), embora nesse ponto de aferição não tenha havido diferença significativa de diâmetro entre os sexos em nenhuma faixa etária.

No ponto de medida mais cranial, a VCI foi estatisticamente mais calibrosa em homens do que em mulheres nas seguintes faixas etárias: 41 a 50 anos (diâmetro médio de 24,6 mm em homens e de 22,7 mm em mulheres; p = 0,01); 51 a 60 anos (diâmetro médio de 24,8 mm em homens e de 22,8 mm em mulheres; p = 0,03); e 61 a 70 anos (diâmetro médio de 24 mm em homens e de 22,4 mm em mulheres; p = 0,03).

Os diâmetros médios (em milímetros) da VCI aferidos nos pontos caudal, médio e cranial em pacientes de ambos os sexos nas diferentes faixas etárias estão apresentados, respectivamente, nas Figuras 4, 5 e 6.

**Figura 4:** Diâmetros médios da veia cava no ponto de aferição caudal em ambos os sexos e nas faixas etárias correspondentes. *p = 0,003; *t* de Student. †p < 0,001; *t* de Student. ‡p < 0,001; Kruskal-Wallis. §p = 0,02; análise de variância.

**Figura 5:** Diâmetros médios da veia cava no ponto médio de aferição em ambos os sexos e nas faixas etárias correspondentes. * e † p<0,001; análise de variância.

**Figura 6:** Diâmetros médios da veia cava no ponto de aferição cranial em ambos os sexos e nas faixas etárias correspondentes. *p = 0,01; teste *t* de Student. † e ‡ p = 0,03; teste *t* de Student. §p = 0,83; Kruskal-Wallis. || p = 0,06; Kruskal-Wallis.

Os diâmetros médios aferidos nos pontos cranial, médio e caudal são, respectivamente, 22,9 mm, 22,7 mm e 23 mm na faixa etária mais baixa (19 a 40 anos) e 21,6 mm, 19,1 mm e 20,6 mm na faixa etária mais alta (81 a 92 anos) em mulheres. No sexo masculino, os diâmetros médios para a faixa etária mais baixa foram, respectivamente, 22,4 mm, 22,8 mm e 22,5 mm e, na faixa etária mais alta, 22,4 mm, 18,5 mm e 19,9 mm. A taxa de variação dos diâmetros médios nos três pontos da VCI está apresentada nas Figuras 7, 8 e 9.

**Figura 7:** Taxas de variação de diâmetro no ponto de aferição caudal, nos sexos masculino e feminino. *p = 0,54; teste *t* de Student.

**Figura 8:** Taxas de variação de diâmetro no ponto médio de aferição, nos sexos masculino e feminino. *p = 0,10; teste *t* de Student.

**Figura 9:** Taxas de variação de diâmetro no ponto de aferição cranial, nos sexos masculino e feminino. *p = 0,48; teste *t* de Student.

Apesar de terem sido identificados diâmetros menores tanto em mulheres quanto em homens mais velhos, a taxa de variação de diâmetros nos pontos cranial, médio e caudal foi semelhante entre os sexos (p = 0,54, p = 0,10 e p = 0,48).

## DISCUSSÃO

O primeiro FVC, modelo de Mobin Uddin, foi desenvolvido em 1969. Desde então, esses filtros sofreram diversas modificações que aumentaram sua eficiência e diminuíram a incidência de complicações^21^^-^23.

O FVC escolhido deve se adequar ao diâmetro da VCI do paciente, a fim de evitar complicações como: perfuração da VCI (Figura 10) ou de estruturas adjacentes, migração do local de implante, trombose do filtro ou embolia pelo próprio dispositivo^8^^-^14. Atualmente, estão disponíveis pelo menos 14 modelos de FVC no Brasil, cujas características, incluindo compatibilidade com o diâmetro da VCI, estão expostas na Tabela 3.

**Figura 10:** Filtro de veia cava perfurando a veia cava inferior. (A) Tomografia computadorizada em corte axial; (B) Tomografia computadorizada em corte coronal. Ponta da seta: hastes fixadoras do filtro perfurando a parede da veia cava. Fonte: arquivo pessoal dos autores.

**Tabela 3:** Tipos de filtros de veia cava e suas características.

**Produto**	**País**	**Produtor**	**Material**	**Forma**	**Diâmetro**	**Remoção**
**GREENFIELD®**	EUA	Boston	Aço/titânio	Cônica	≤ 28mm	Permanente
**GUNTHER®**	EUA	Cook	Coni-Chrome	Cônica	≤ 30mm	≤ 3 semanas*
**BIRD’S NEST®**†	EUA	Cook	Coni-Chrome	Ninho	35-40mm	Permanente
**CELECT®**	EUA	Cook	Coni-Chrome	Cônica	≤ 30mm	≤ 3 meses*
**VENATECH CONVERTIBLE®**	França/EUA	BBraun	Phynox	Cônica	≤ 32mm	≤ 4 semanas*
**VENATECH RETRIEVABLE®**	França/EUA	BBraun	Phynox	Cônica	14-28mm	≤ 12 semanas*
**VENATECH LP®**	França/EUA	BBraun	Phynox	Cônica	28-35mm	Permanente
**VENATECH LGM®**	França/EUA	BBraun	Phynox	Cônica	≤ 28mm	Permanente
**TEMPOFILTER II®**	França/EUA	BBraun	Phynox	Cônica	≤ 32mm	Permanente
**SIMON®**	Reino Unido	Bard	Nitinol	Cogumelo	≤ 32mm	≤ 12 semanas*
**TRAPEASE®**	EUA	Cordis	Nitinol	Trapezoide	≤ 30mm	Permanente
**OPTEASE®**	EUA	Cordis	Nitinol	Trapezoide	≤ 30mm	≤ 21dias*
**ALN®**	França	ALN	Aço	Cônica	≤ 32mm	≤ 25 meses*
**ELLA®**	Rep.Tcheca	ELLA	Aço	Cônica	18-35mm	≤ 12 dias*

* Tempo indicado pelo fabricante como limite máximo para recuperação do filtro;

† Sem registro para uso no Brasil. Fonte: Agência Nacional de Vigilância Sanitária (ANVISA)^24^.

A topografia clássica para implante dos FVCs é o segmento infrarrenal, em que se recomenda que a porção superior do filtro fique localizada imediatamente abaixo da veia renal mais caudal (coincidindo com o ponto cranial de aferição de diâmetro neste estudo), de modo que, na ocorrência de uma eventual retenção de trombos obstruindo o filtro, a drenagem renal não seja prejudicada.

Após revisão da literatura, não foram identificados estudos que demonstrassem a tendência de pacientes mais velhos apresentarem menores diâmetros da VCI em seu segmento infrarrenal, como demonstrado nesta pesquisa, ou que discutissem a possível correlação da redução de diâmetro com o desenvolvimento de complicações tardias dos FVCs.

Em 2010, Masugata et al.^15^ publicaram resultados de avaliação do diâmetro do segmento intrapericárdico da VCI e demonstraram que, em pacientes mais velhos, a tendência é que haja uma contração das paredes da cava, levando à progressiva diminuição do lúmen nessa topografia. Eles sugeriram que tais variações ocorreriam devido à diminuição da pressão do átrio direito e da complacência da VCI com o passar da idade.

Neste estudo, a distribuição dos pacientes foi feita de acordo com as décadas de vida, semelhante ao trabalho de Masugata et al.^15^. Entretanto, como a indicação de exames de imagem é mais frequente em pacientes mais velhos, para que tivéssemos grupos com quantidades comparáveis de pacientes, aqueles com idade entre 19 e 40 anos foram agrupados. Tanto no estudo de Matsugata et al.^15^ quanto na presente pesquisa, a relação entre diâmetros da VCI e a idade dos pacientes foi feita em um único momento de observação. A metodologia ideal, inviável pelas inúmeras limitações, envolveria acompanhar as modificações do diâmetro venoso ao longo de décadas em um número significativo de pacientes.

Apesar dessas limitações, as conclusões de ambos os estudos convergem para uma tendência à redução do diâmetro da VCI conforme o paciente envelhece. Esse fenômeno é oposto ao que ocorre com a aorta abdominal, cujo diâmetro tende a aumentar, como já demonstrado em uma pesquisa anterior publicada por nosso grupo^25^.

Durante o envelhecimento, a diminuição de colágenos e os efeitos dos radicais livres certamente são sistêmicos e se manifestam tanto sobre as veias quanto sobre as artérias, porém com consequências opostas nesses grandes vasos abdominais^26^^-^29. Essa diferença provavelmente é devida ao fato de que, enquanto o sistema arterial tem a sua fisiologia mais baseada em “pressão”, o sistema venoso tem o seu funcionamento baseado em “capacitância”^30^. Conforme o paciente envelhece, há uma maior predisposição à estase sanguínea periférica, influenciando inclusive no surgimento de insuficiência venosa^31^. Dessa forma, em teoria, a VCI passaria a armazenar um volume sanguíneo progressivamente menor. Com menor distensão e perda da elasticidade parietal, o seu diâmetro progressivamente diminuiria; porém, ainda faltam estudos que comprovem essas teorias.

Embora cada filtro seja capaz de se adaptar a diferentes diâmetros venosos, essa capacidade adaptativa tem um limite e relaciona-se à adaptação do diâmetro do filtro no momento do seu implante. A forma como a estrutura do dispositivo se acomodaria à eventual diminuição do calibre de uma veia com paredes cada vez menos elásticas é uma incerteza e isso pode estar associado à perfuração tardia da VCI e ao consequente risco de lesão de estruturas adjacentes^9^^-^11^,^^14^.

De acordo com nossos resultados, a maioria dos filtros é compatível com os diâmetros infrarrenais das VCIs de homens e mulheres adultos. Entretanto, a progressiva redução dos diâmetros da VCI pode dificultar a compatibilidade com os modelos Bird’s nest® (Cook Medical, Bloomington, IN, EUA) e VenaTech® LP (B. Braun Sharing Expertise, Melsungen, Alemanha), por serem indicados para diâmetros acima de 28 mm.

Muitas vezes, o filtro implantado é o “disponível”, por exemplo, aquele liberado pelo plano de saúde ou acessível em determinado hospital público, o que pode aumentar os riscos de complicações. Por exemplo, se de acordo com as instruções do fabricante, um determinado filtro for compatível com cavas com diâmetro entre 28 e 35 mm (Tabela 3) for implantado em uma mulher com menos de 40 anos de idade, como o diâmetro no ponto médio de aferição do segmento infrarrenal para esse perfil de paciente foi, em média, de 22,8 mm, o risco de complicações agudas já seria potencialmente alto. Em teoria, o risco seria ainda maior conforme ocorre o envelhecimento da paciente, considerando que, durante as próximas quatro décadas de vida, ela pode ter o diâmetro da cava reduzido em 15,9%, o que aumentaria o risco de perfuração da VCI.

A implicação prática do presente estudo ratifica que, sempre que possível, a escolha do modelo de FCV a ser implantado recaia sobre os filtros removíveis e ressalta a importância da avaliação individual do diâmetro da cava antes do implante^32^.

Entre as limitações do estudo, estão o seu caráter retrospectivo; por exemplo, não foi possível recuperar dados sobre a altura e o peso dos pacientes que, hipoteticamente, poderiam estar relacionados a variações do diâmetro da VCI. Além disso, embora tenha sido realizado um cálculo de tamanho amostral e tenhamos obtido um número total de pacientes superior à amostra mínima, a distribuição do número de pacientes entre as faixas etárias ocorreu por conveniência, de acordo com a disponibilidade dos exames realizados. Sugere-se o aprofundamento de pesquisas sobre o tema.

## CONCLUSÃO

O diâmetro do segmento infrarrenal da VCI foi menor em pacientes com idade mais avançada, tanto em homens quanto em mulheres, e o sexo dos pacientes não influenciou significativamente a taxa de variação do diâmetro.

A maioria dos modelos de FVC é compatível com os diâmetros infrarrenais das VCIs de homens e mulheres adultos entre 20 e 92 anos.
